# Women’s empowerment indicators and short- and long-acting contraceptive method use: evidence from DHS from 11 countries

**DOI:** 10.1186/s12978-022-01532-5

**Published:** 2022-12-06

**Authors:** Kenneth Setorwu Adde, Edward Kwabena Ameyaw, Kwamena Sekyi Dickson, Jones Arkoh Paintsil, Olanrewaju Oladimeji, Sanni Yaya

**Affiliations:** 1 Department of Population and Health, University of Cape Coast, Cape Coast, Ghana; 2 Institute of Policy Studies and School of Graduate Studies, Lingnan University, Tuen Mun, Hong Kong; 3 L & E Research Consult Ltd, Upper West Region, Ghana; 4 Department of Economic Studies, School of Economics, University of Cape Coast, Cape Coast, Ghana; 5 Department of Public Health, Walter Sisulu University, Mthatha, Eastern Cape 5099 South Africa; 6 The George Institute for Global Health, Imperial College London, London, UK; 7 School of International Development and Global Studies, University of Ottawa, Ottawa, ON Canada

**Keywords:** Women empowerment, Contraception, Sub-saharan Africa, Reproductive health

## Abstract

**Background:**

With a population of about 1.1 billion, sub-Saharan Africa is projected to overtake Eastern, Southern and Central Asia to become the most populous region by 2060. One effective approach for slowing this rapid population growth is the use of modern contraception and this may be short-acting or long acting. Previous studies have explored the association between women empowerment indicators contraception use, however, there is limited evidence on how women empowerment indicators associate with type of contraception. Hence the present study investigated the association between women empowerment indicators and type of contraception used by women in 11 sub-Saharan African countries.

**Methods:**

We utilised Demographic and Health Survey data of 22,637 women from 11 countries, collected between 2018 and 2021. The outcome variable was type of contraception used. Descriptive and inferential analyses were executed. The descriptive analysis reflected women empowerment indicators and the proportion of women using contraceptives. Multinomial logistic regression was considered for the inferential analysis. The results for the multinomial logistic regression were presented as adjusted odds ratios (aORs) along with the respective 95% confidence intervals (CIs) signifying precision. The sample weight (wt) was used to account for the complex survey (svy) design. All the analyses were done with Stata version 13 and SPSS version 25.

**Results:**

The study showed that on the average, 15.95% of the women do not use modern contraceptives, whilst 30.67% and 53.38% use long-acting and short-acting contraceptives respectively. The adjusted models showed that women who were working had higher odds of using long-acting (aOR = 1.44, CI 1.28–1.62) and short-acting (aOR = 2.00, CI 1.79–2.24) methods compared with those who were not working. The analysis revealed higher likelihood of long-acting method use among women with high decision-making capacity (aOR = 1.27, CI 1.09–1.47) compared with women with low decision-making capacity. Women with medium knowledge level had a higher likelihood (aOR = 1.54, 1.09–2.17) of using long-acting methods than their counterparts with low knowledge level.

**Conclusion:**

Our findings show that most women in the 11 countries use modern contraceptives, however, different empowerment indicators align with different contraceptive type. It therefore behoves governments of the studied countries to review current interventions and embrace new ones that are more responsive to the peculiar contraception needs of empowered and non-empowered women.

## Plain Language summary

By 2060, sub-Saharan Africa is expected to have more population than Southern, Eastern, and Central Asia. Modern contraception is identified to be effective in reducing population growth. There is limited evidence on the association between women empowerment indicators and the type of contraception used by women in sub-Saharan Africa, either short or long-acting methods. Hence, this study investigated the relationship between women empowerment indicators and the type of contraception used by women in 11 sub-Saharan African countries. We performed both descriptive and inferential analyses. The descriptive analysis, being frequencies and percentages, focused on women empowerment indicators and the proportion of women who use contraception. The inferential analysis was multinomial logistic regression. The findings showed that 15.95% of women do not use modern contraception, however, 30.67% and 53.38% were using long-acting and short-acting contraception, respectively. Women who worked had higher odds of using long-acting and short-acting methods than those who did not work. Women with high decision-making capacity had high likelihood of using long-acting methods than women with low decision-making capacity. Women with medium knowledge level had a higher likelihood of using long-acting methods than their counterparts with low knowledge level. The study highlights the need for governments in the studied countries to review current interventions and adopt new ones to make the interventions more responsive to the contraception needs of women, irrespective of their empowerment status.

## Background

Sustained exponential population growth compromises efforts garnered toward the achievement of the Sustainable Development Goals [1]. The rapid rate of population growth in sub-Saharan Africa (SSA) is anticipated to continue till the end of the twenty-first century [2]. The sub-region currently has a population of about 1.1 billion and it is projected to outnumber Eastern, Southern and Central Asia to become the most populous region by 2060 [2]. One effective approach for slowing this rapid population growth is the use of modern contraception or family planning. As a result, target 3.7 of the United Nations’ Sustainable Development Goals (SDGs) aims at ensuring universal access to sexual and reproductive healthcare services, including family planning, information and education, and the integration of reproductive health into national strategies and programs [1]. Contraception use is associated with enhanced economic growth, poverty decline and improved family productivity through fertility reduction and its positive contribution towards child survival and maternal wellbeing [3, 4].

Contraceptives may be classified into short-acting and long-acting reversible methods. Long-acting reversible methods refer to a group of contraception methods with high degree of reliability and effectiveness, and do not require users to adhere to any routine regimen [5]. These methods are convenient and cost effective [5]. Short-acting methods, on the other hand, constitute effective user-dependent contraception methods which need to be taken on a routine regimen such as daily, weekly or monthly [6]. Short and long-acting contraceptives have their peculiar merits and demerits. For instance, long-acting methods are cost effective, have low failure rate and are more tolerable, however, they usually require a healthcare provider’s assistance unlike short-acting methods [7–9]. Factors such as fear of side-effects, disapproval by male partner, and social/cultural norms about contraception use affect it’s utilisation [10]. Women’s choices about the type of contraception to use might be informed by their knowledge on side effects, effectiveness or otherwise, availability and accessibility [11, 12].

Patriarchal rule compromise women’s ability to enjoy their fundamental reproductive health rights in some parts of SSA [13, 14]. Besides, some family planning initiatives in SSA could not achieve their intended objective because they failed to consider the power relations between women and their partners [13]. Women in some households across SSA cannot make any decision on whether to use contraception or otherwise as well as the type of contraception to use. Greno and Saikia [13] therefore contend that women empowerment should not be taken lightly in fertility and contraception discourses. Though diverse indicators have been used in gauging women empowerment, several scholars agree that decision making capacity, knowledge level, employment status and disposition towards wife beating are indispensable indicators for measuring women empowerment [15–17]. These indicators have been used extensively to investigate utilization of a wide array of health services [18, 19]. To this end, the objective of this study is to investigate the association between women empowerment indicators and the type of contraception used in 11 SSA countries, in order to proffer pragmatic recommendations that would propel the sub-region to meet SDG target 3.7. Additionally, the study will reveal the association between various empowerment indicators and types of contraception (either long or short term) used by women in the included countries, thereby offering basis to guide reproductive health interventions.

## Methods

### Data source and sampling

This study made use of Demographic and Health Survey (DHS) data of 22,637 women from 11 SSA countries. Our inclusion criteria was that the SSA country must have had DHS between 2018 and 2021 and should have the variables of interest. DHS uses a two-stage stratified cluster sampling approach to select nationally representative samples of women of reproductive age (15–49 years). The first stage involves a listing of primary sampling units (PSUs), or enumeration areas (EAs), and are generally obtained from the latest national census of the respective countries [20]. Each EA is further subdivided into standard size segments and a sample of predetermined segment is selected randomly with probability proportional to the number of households in each EA. In the second stage, households are systematically recruited by surveying them from a list of previously enumerated households in each EA [20]. DHS datasets can be accessed from https://dhsprogram.com/. The Strengthening the Reporting of Observational Studies in Epidemiology (STROBE) statement was relied upon while conducting this study and writing the manuscript.

**Table 1:** Basic descriptive information of study sample

Country	Year of DHS survey	Frequency	Proportion not using modern contraceptive (%)	Proportion using short term modern contraceptive (%)	Proportion using long term modern contraceptive (%)
Benin	2018	1625	23.14	31.31	45.55
Cameroon	2018	1466	26.44	54.81	18.75
Gambia	2020	1285	9.96	57.43	32.61
Guinea	2018	722	40.70	38.70	20.60
Liberia	2020	1265	4.99	75.87	19.14
Mali	2018	1252	6.02	49.26	44.72
Mauritius	2021	1072	10.60	72.34	17.06
Nigeria	2018	3795	33.81	40.04	26.15
Rwanda	2020	4664	11.53	43.68	44.79
Sierra Leone	2019	1922	2.66	63.15	34.19
Zambia	2018	3569	6.47	76.88	16.66
Total	–	22,637	15.95	53.38	30.67

### Study variables and measurements

#### Outcome variable

In this study, the outcome variable was contraceptive method which was derived from the women’s responses when they were asked whether they were currently doing or using something to delay or avoid pregnancy. The outcome variable had three outcomes; long-acting methods, short-acting methods and no method or not using contraception. Long-acting methods are those that last longer than 3 months, which include the IUD, implants, female sterilization and male sterilization. Short acting methods include pills, injections, condoms or diaphragm/foam/jelly. Women who cited no method, abstinence, withdrawal or lactational amenorrhoea were coded as not using contraception/no method. Those who had never had sex were excluded from the study.

#### Explanatory variables

The main independent variable was women empowerment indicators. In conformity with previous studies [21–23], four empowerment indicators were used, namely; (1) Labour force participation (not working, working), (2) Acceptance of wife beating (neglect of child, going out without permission, arguing with husband/partner, burning of food, refusal to have sex with husband/partner), (3) Decision making power (measured by the person who makes decisions concerning respondent’s health care, house earning, household purchase, and visiting family members), (4) Knowledge level (measured by frequency of listening to radio, reading newspaper/magazine, watching television, and educational level). Both decision making power and acceptance of wife beating were categorised into low, middle and high in accordance with some earlier studies [22, 23]. Other independent variables included age (15–19, 20–24, 25–29, 30–34, 35–39, 40–44, 45–49); place of residence (urban or rural); marital status (married or living with partner) and partner’s education (no education, primary, secondary, higher).

### Data analysis

Descriptive and inferential analyses were done. The descriptive analysis reflected results on women empowerment indicators and the proportion using contraceptives. Multinomial logistic regression was employed for the inferential analysis and this is because the outcome variable was nominal in nature with three outcomes (not using contraceptive, using short acting contraceptive and using long-acting contraceptive). The results for the multinomial logistic regression were presented as adjusted odd ratios (aOR) along with the respective 95% confidence intervals (CIs) signifying precision, using SPSS. The sample weight (wt) was used to account for the complex survey (svy) design and generalizability of the findings. All the analyses were done with Stata version 13 and SPSS version 25.

### Ethics

We used publicly available secondary data for the analysis. Due to that, no additional ethical approval was required beside the ethical protocols followed by the DHS Program. Both written and verbal ethical approval were sought from research participants, as reported by the DHS Program.

## Results

On the average, the prevalence of long-acting modern contraceptive was 30.7% with Zambia recording the lowest (16.7%) and Benin recording the highest (45.6%). The average prevalence of short term modern contraceptive use was 53.4% with Benin recording the lowest (31.3%) and Zambia recording the highest (76.9%). The average prevalence of women who were not using modern contraceptive was 16% with Sierra Leone recording the lowest prevalence (2.7%) of women not using contraceptive and Guinea recording the highest prevalence (40.7%) of women not using modern contraceptive (see Table 1).

Table 2 shows that 21.9% of the women were aged 30–34 and 54.2% of them resided in rural areas. For 34.2% of the women, their partners had primary education and 83.8% of the women were married. It was also observed that 63% of the women had a medium level of knowledge, majority of women (66.5%) had low acceptance of wife beating, about 54.1% have a high decision-making power and 79% of the women are working.

**Table 2:** Background characteristics of respondents and type of contraceptive

Variable	Weighted n	Weighted %	Not using Modern contraceptive (%)	Using short term modern contraceptive (%)	Using long term modern contraceptive (%)
*Age*					
15–19	668	2.95	16.78	58.33	24.90
20–24	2980	13.17	13.37	57.48	29.14
25–29	4908	21.68	15.38	54.20	30.42
30–34	4956	21.89	15.02	52.66	32.32
35–39	4785	21.14	16.44	52.56	31.01
40–44	2859	12.63	17.48	50.73	31.79
45–49	1481	6.54	21.20	50.44	28.36
*Place of residence*					
Urban	10,360	45.77	18.30	54.90	26.80
Rural	12,277	54.23	13.97	52.10	33.93
*Partner’s educational level*					
No education	5306	23.44	13.67	50.74	35.59
Primary	6707	29.63	14.93	52.02	33.05
Secondary	7734	34.17	16.74	56.70	26.55
Higher	2890	12.77	20.35	52.54	27.11
*Marital status*					
Married	18,980	83.84	16.51	54.22	29.27
Cohabiting	3657	16.16	13.04	49.04	37.93
*Decision-making power*					
Low	3947	17.44	14.77	53.37	31.86
Medium	6453	28.51	14.48	54.39	31.86
High	12,237	54.06	17.10	52.86	30.03
*Women’s knowledge level*					
Low	3023	13.36	12.13	54.63	33.24
Medium	14,250	62.95	15.75	53.50	30.75
High	5364	23.69	18.61	52.38	29.00
*Labour force participation*					
Not working	4674	20.65	9.59	63.51	26.89
Working	17,963	79.35	17.60	50.75	31.65
*Acceptance of wife beating*					
Low	15,059	66.53	17.48	53.21	29.31
Medium	3625	16.01	11.99	54.53	33.48
High	3953	17.46	13.75	53.01	33.25

Table 3 reports findings from the multinomial logistic regression modelling. With no contraception method as the base, we noted that women who were working had higher odds of using long-acting methods compared with those who were not working (aOR = 1.44, CI 1.28–1.62). Those working had higher likelihood of using short acting methods than their counterparts who were not working (aOR = 2.00, CI 1.79–2.24). The analysis revealed higher likelihood of long-acting method use among women with high decision-making capacity (aOR = 1.27, CI 1.09–1.47) compared with women with low decision-making capacity.

Women with medium knowledge level had a higher likelihood (aOR = 1.54, 1.09–2.17) of using long-acting methods than their counterparts with low knowledge level. We observed that women with high acceptance of wife beating had higher odds of using long-acting methods as compared to women with low acceptance of wife beating (aOR = 1.11, CI 1.01–1.22). A higher odd was observed for short acting method use among women with higher acceptance of wife beating (aOR = 1.14, CI 1.04–1.25) as compared to their counterparts with low acceptance. With regards to age, the likelihood of using long-acting methods increased with age. However, the odds of using short acting methods was highest among women aged 35–39 (aOR = 1.37, CI 1.17–1.61).

**Table 3:** Multinomial logistic regression of long-acting contraceptive uptake in 11 SSA countries

Variable	aOR	[95% CI]	aOR	[95% CI]	aOR	[95% CI]	aOR	[95% CI]
	Long-acting method vs. none		Short acting method vs. none		Long-acting method vs. none		Short acting method vs. none	
*Occupation*								
Not working	Ref.	Ref.	Ref.	Ref.	Ref.	Ref.	Ref.	Ref.
Working	1.35***	1.20–1.51	2.06***	1.85–2.29	1.44***	1.28–1.62	2.00***	1.79–2.24
*Decision making capacity*								
Low	Ref.	Ref.	Ref.	Ref.	Ref.	Ref.	Ref.	Ref.
Medium	0.76***	0.68–0.85	0.85***	0.76–0.94	0.88*	0.79–0.99	0.92	0.83–1.03
High	1.20**	1.04–1.41	1.21***	1.05–1.39	1.27***	1.09–1.47	1.25***	1.09–1.44
*Knowledge level*								
Low	Ref.	Ref.	Ref.	Ref.	Ref.	Ref.	Ref.	Ref.
Medium	1.51***	1.31–1.75	1.55***	1.35–1.77	1.54*	1.09–2.17	1.25	0.90–1.72
High	1.19***	1.08–1.31	1.19***	1.08–1.31	1.14	0.94–1.39	1.04	0.87–1.24
*Acceptance of wife beating*								
Low	Ref.	Ref.	Ref.	Ref.	Ref.	Ref.	Ref.	Ref.
Medium	1.07	0.95–1.21	0.99	0.89–1.11	1.09	0.96–1.23	1.03	0.92–1.15
High	1.10*	1.00–1.21	1.13**	1.04–1.24	1.11*	1.01–1.22	1.14***	1.04–1.25
*Age*								
15–19					Ref.	Ref.	Ref.	Ref.
20–24					0.73*	0.55–0.98	0.92	0.72 1.19
25–29					1.34**	1.10–1.63	1.31***	1.11–1.57
30–34					1.44***	1.20–1.71	1.28***	1.09–1.51
35–39					1.60***	1.35–1.91	1.37***	1.17–1.61
40–44					1.52***	1.28–1.81	1.32***	1.13–1.54
45–49					1.31**	1.09–1.58	1.18*	1.11–1.39
*Marital status*								
Married					Ref.	Ref.	Ref.	Ref.
Living with partner					0.62***	0.55–0.72	0.92	0.82–1.03
*Partner’s education*								
No education					Ref.	Ref.	Ref.	Ref.
Primary					1.21	0.94–1.34	0.92	0.78–1.08
Secondary					0.93	0.78–1.09	0.79***	0.68–0.92
Higher					0.90	0.78–1.05	0.98	0.85–1.12
*Residence*								
Urban					Ref.	Ref.	Ref.	Ref.
Rural					0.86***	0.78–0.94	0.99	0.91–1.07

Exponentiated coefficients; Ref = reference category; *p < 0.05, **p < 0.01, ***p < 0.001

Cohabiting women had lower odds of using long-acting methods (aOR = 0.62, CI   0.55–0.72) and short acting methods (aOR = 0.92, CI 0.78–1.08). Compared to urban women, rural women had lower odds of using long-acting method (aOR = 0.86, CI 0.78–0.94). Rural women also had a lower likelihood of utilizing short acting methods as compared to urban women (aOR = 0.99, CI 0.91–1.07).

## Discussion

Previous evidence have illustrated that women’s empowerment indicators such as labor force participation, decision making capacity, knowledge and disposition towards wife beating affect women’s ability to enjoy their fundamental reproductive health rights and utilise their preferred health services [13, 21, 24]. In spite of this, the relationship between women empowerment indicators and women’s choice of contraceptives has not received much consideration. To this end, the present study investigated the association between women empowerment indicators and its relationship with the type of contraception women use.

The study revealed that women who were working had increased likelihood of using short-acting contraceptive methods. Another study from Nepal reported that women who work have lower likelihood of using long-acting methods, suggesting a higher inclination towards short-acting methods [25]. Compared with women who do not work, those who work are likely to have the purchasing power and hence being able to access a wide array of contraceptives of their choice. For instance, a multi-country study on household wealth and contraception use reported that poorer women in Bangladesh and India had higher inclination towards long-acting contraception methods, than the wealthier women [26]. This notwithstanding, other scholars have reported inconsistent findings, possibly due to variation in study design or differences in the socio-demographic characteristics of the samples studied [26, 27].

The analysis revealed higher likelihood of long-acting method use among women with high decision-making capacity. Couples in these 11 countries may be prioritizing long-acting contraception methods because of the advantages of the long-acting methods such as their cost effectiveness and the low failure rate as other authors have reported [7–9].

It was noted that women with medium knowledge level had higher odds of using long-acting methods than their counterparts with low knowledge level. Meanwhile, women with high acceptance of wife beating had higher likelihood of using long-acting contraception methods. Women who accept wife beating usually have no or low education, and do not participate much in decision making at the household level [28, 29]. On the part of women with medium level of knowledge, their inclination towards long-acting methods could imply that they consider the merits of these methods weightier compared to the benefits associated with short-acting contraception methods. For instance, long-acting methods do not require shorter interval visit to the health facility or service of a trained healthcare professional as would a short-term contraception method [30]. Hence, these dynamics might account for the observed inclination towards short and long-acting contraception methods by the different groups.

Notably, different empowerment indicators were aligned with either short or long-acting methods, without any clear pattern or consistency in contraception type. These key findings might be indicative of possible structural motivations and barriers that intervene with the various empowerment indicators to determine the type of contraception that are preferred and accessible to the women. Irrespective of a woman’s empowerment status, she is more probable to prioritise a contraception method that is easily accessible. For instance, considering that most long-acting methods require the service of a trained health professional, women with limited access to locations where such a service can be accessed are likely to procure and utilise short-acting contraception methods such as pills and condoms and develop affinity for them as opposed to long-acting methods [12].

All things being equal, long-acting methods should commensurate the availability of facilities and health personnel who will administer these methods [31]. Though our study was limited by its inability to explore whether the methods used are the preferred methods or not, the findings accentuate that beyond women’s empowerment status, structural factors may have to be targeted in ensuring that women are able to access and utilise their preferred methods but not just improvise what the system dictates to them [32].

In addition to the empowerment indicators, a number of socio-demographic characteristics proved relevant in contraception method selection. For instance, the odds of using long-acting methods increased with age. Evidence suggest that motivation for using long-acting methods include long term protection against unintended pregnancies, effectiveness and better child-spacing [12, 33]. Though short-acting methods have competitive advantages, this finding might suggest that as women advance in age, they weigh long-acting methods to be more protective, safe, and reliable as compared with short term contraceptive methods [27].

Compared to urban women, rural women had lower odds of long-acting method use. Contrary to these findings, dominance of long-acting contraception method use among rural women has been reported by some previous studies from China and Guatemala [34, 35]. However, our finding is an embodiment of the contraception use situation of SSA, considering that long-acting methods require trained service providers/health facilities coupled with the extent to which these facilities are skewed towards urban settings in the SSA context [34, 36].

## Strengths and limitations

This study has a number of notable strengths. Firstly, it gauged women empowerment with notable indicators which are widely used. The study also employed rigorous analytical procedure, with representative samples from 11 SSA countries. Besides, unlike previous studies that focused on women empowerment indicators and contraception use in general, this study advances the discourse by investigating the types of contraceptives used with respect to the empowerment indicators. The limitations of the study include the possibility that some women misreported the type of contraception they use, decision making capacity and working status in order to conform to social expectations. Also, the cross-sectional study design makes causal inference between the women empowerment indicators and type of contraception impossible. Despite these limitations, the study is a true reflection of women empowerment indicators and type of contraception used by women in the 11 SSA countries studied.

## Conclusion

The study showed that on the average 15.95% of women in the 11 surveyed countries do not use modern contraceptives, whilst 30.67% and 53.38% use long-acting and short-acting contraceptives respectively. The study showed a generally significant association between the empowerment indicators and type of contraception. Meanwhile, we noticed inconsistent association between the four empowerment indicators and type of contraception women use. For instance, women who were working had increased likelihood of using short-acting contraceptive methods whereas lower likelihood of long-acting method use was noted among women with high decision-making capacity. It therefore behoves governments of the studied countries to review current interventions and embrace new ones that are tailored to meet the peculiar contraception needs of empowered and non-empowered women.

## Data Availability

Data for this study were sourced from Demographic and Health surveys (DHS) and available here: http://dhsprogram.com/data/available-datasets.cfm.
